# Integration of dentistry and forensic odontology for a structured identification system and border control

**DOI:** 10.1080/20961790.2020.1842155

**Published:** 2021-02-08

**Authors:** Emilio Nuzzolese

**Affiliations:** Department of Sciences of Public Health and Pediatrics, University of Turin, Turin, Italy

Dear Editor,

The increase in migration across countries has led to a range of problems in public health, border control, traveller and deceased identification, age assessment on unaccompanied minors, and identification of dead migrants. A review of these procedures in Italy highlights the need for a wider and centralized collection of antemortem and postmortem data as of the quality of postmortem data collection [1].

Non-European Union (EU) nationals entering Europe are controlled by passports and fingerprints. Illegal migrants are without identification documents and for this reason they have to follow a specific legal path in order to issue proper identification documents, evaluate the juridical condition and the rights of undocumented migrants, as well as public health assessments. Migrants are taken to a hosting center where they are lodged until they are identified. They receive humanitarian assistance and health care during their stay at the hosting center, including general health evaluations, medical examinations, X-rays, blood chemistry tests, and microbiological analysis. Dentistry is not included in the general health evaluation, although dental care often seems to be requested by migrants (depending on their length of stay at the hosting centers).

Age estimation of unaccompanied minors arriving at the borders of Italy is not performed using a national standardized protocol. Age estimation procedures can include physical examination, psycho-social interview and the wrist X-ray of the not writing hand. In some cases, dental age estimation methods are applied but are not systematically included in the assessment [2–5].

Missing and unidentified dead migrants face further challenges. Standards in the forensic examination and postmortem collection are not sufficient to satisfy basic quality criteria for the management of the dead and their rights. As a result, the “Missing Migrants Project” estimates that there have been 17 124 migrant deaths and disappearances since 2014 and over 60% of these victims remain unidentified [6,7]. Visual identification is the most common method used to identify dead migrants, although this method has well known to have huge limitations, including the presence of at least one family member of the dead migrant, which in most cases is not available.

Italy has the capacity of collecting identifying data respecting best practices in human identification and the human rights of the dead [6]. Different from other medical disciplines, dentistry can involve not only oral health care and assistance, but also forensic applications in the personal identification of both living and dead individuals. When only postmortem dental data are available, a generic biological profile can be assessed to narrow the search and investigation; when antemortem dental data are, or become available, a positive identification can be achieved through comparison and reconciliation. The author’s experience in volunteering as dentist in hosting centers, and as forensic odontologist during identification autopsies of dead migrants recovered from the sea, highlights the need to consider the inclusion of community dentistry as part of the general health evaluation and assistance of migrants [8] and the systematic involvement of forensic odontology in the postmortem collection, which is still limited to a dental examination, rather than a complete dental autopsy.

The clinical evaluation of migrants at the EU border could follow the *dental fitness* scores model suggested for military personnel by the North Atlantic Treaty Organization (NATO) agreement 2466 (STANAG2466) [9] and would allow a comprehensive health assessment and wellbeing of migrants. Several researches have confirmed that migrants coming from developing countries have substantially poor oral health with negative effects on the quality of life [10–12] and should receive essential dental treatments as part of the essential healthcare, as outlined by international conventions and treaties [13]. The right to health is recognized in the inherent dignity of the equal and inalienable human right: article 25 of the Universal Declaration of Human Rights states that “Everyone has the right to a standard of living adequate for the health and wellbeing of himself and of his family” [14].

Dental clinical assessment and dental assistance would allow the creation of migrant dental records, archived in a traditional database or in the health electronic record (EHR) of the patient which could be also integrated with a blockchain-based architecture and interoperable design when personal identification of a living or a dead migrant is required [15,16]. In some cases, illegal migrants attempt to enter using an alias or false, incomplete, or altered fingerprints. In these cases, the judicial authorities could rely on dental data and age verification using dental methods [17], which could be used to confirm the identity.

Dental clinical assessment and dental assistance should be provided to migrants coming from developing countries when crossing border. Oral health care professionals could offer oral treatments of community dentistry on a voluntary basis. This would allow the creation of a national dental database, which may be disclosed to the authorities in case of crime investigations or identification. Forensic odontology should be methodological in all identifying procedures involved in border control and age estimation as in unidentified human remains. This would be achieved through community dentistry programmes and humanitarian forensic odontology organizations involved in human identification and human rights [18,19].


Emilio Nuzzolese
*Department of Sciences of Public Health and Pediatrics* *University of Turin* *Turin, Italy* emilio.nuzzolese@unito.it
http://orcid.org/0000-0001-9706-1106

